# Can *Clethra barbinervis* Distinguish Nickel and Cobalt in Uptake and Translocation?

**DOI:** 10.3390/ijms160921378

**Published:** 2015-09-07

**Authors:** Tsuyoshi Yamaguchi, Rie Tomioka, Chisato Takenaka

**Affiliations:** Graduate School of Bioagricultural Sciences, Nagoya University, Furo-cho, Chikusa-ku, Nagoya 464-8601, Japan; E-Mails: tomiokar@agr.nagoya-u.ac.jp (R.T.); chisato@agr.nagoya-u.ac.jp (C.T.)

**Keywords:** accumulation, split-root experiment, sulfur, translocation, uptake, competition, *Clethra barbinervis*

## Abstract

*Clethra barbinervis* Sieb. et Zucc. accumulates Nickel (Ni) and Cobalt (Co) at high concentrations., We hypothesized that *C. barbinervis* cannot distinguish between Ni and Co because of the similar chemical properties of these two elements. To confirm this hypothesis and understand the role of these elements in *C. barbinervis*, we conducted a hydroponic split-root experiment using Ni and Co solutions. We found that the bioconcentration factor (BCF; metal concentration of each tissue/metal concentrations of each treatment solution) of Ni and Co did not significantly differ in the roots, but the BCF for Co was higher than that for Ni in the leaves. The leaves of *C. barbinervis* accumulated Ni or Co at high concentrations. We also found the simultaneous accumulation of Ni and Co by the multiple heavy metal treatments (Ni and Co) at high concentrations similar to those for the single treatments (Ni or Co). Elevated sulfur concentrations occurred in the roots and leaves of Co-treated seedlings but not in Ni. This result indicates that S was related to Co accumulation in the leaves. These results suggest that *C. barbinervis* distinguishes between Ni and Co during transport and accumulation in the leaves but not during root uptake.

## 1. Introduction

*Clethra barbinervis* Sieb. et Zucc. is a tree species belonging to the family Clethraceae in the order Ericales that naturally grows in the hills and mountains of temperate East Asia [1]. *C. barbinervis* has been recognized as a plant accumulating heavy metals such as Ni, Co, Cd, Zn, and Mn in leaves [2], and particularly accumulates Ni and Co at high concentrations [3]. Ni and Co belong to the Fe group of elements and their chemical properties are similar [4]. Although Ni is essential for the function of plant ureases, the levels required are low [4]. An essential role for Co in plants has not been demonstrated, but Co promotes the growth of some plant species by enhancing the growth of root symbionts that require Co for N_2_ fixation [5]. An interesting observation regarding the accumulation of Ni and Co was reported following an analysis of *C. barbinervis* leaves taken from two different geological sites [3]. Whereas *C. barbinervis* accumulated Ni in serpentine soil, the tree accumulated Co in schist soil at high concentrations; although the concentrations of both Ni and Co were higher in the serpentine soil than in the schist soil [3]. Because of the similarities of Ni and Co, we questioned whether *C. barbinervis* could distinguish between Ni and Co during absorption and accumulation. Co frequently interacts antagonistically with Ni in plants [6,7], and antagonistic interactions between Ni and Co have been observed for *Alyssum bertolonii* [8] and *Berkheya coddii* [9]. The antagonistic relationship may represent a competition for transporters or binding ligands, or their alternative functions between Ni and Co. Therefore, we hypothesized that *C. barbinervis* absorbs and accumulates Ni and Co without distinction. To test our hypothesis, a split-root experiment using solutions of Ni and/or Co was conducted [10,11,12,13]. Subsequently, S assimilation and the production of S-containing compounds such as glutathione and phytochelatin play critical roles in the accumulation and homeostasis of trace elements in plants [14]. Additionally, it was reported that S co-localize and have a positive correlation with Ni or Co in leaves [15,16,17]. Therefore, to examine the mechanism of absorption and accumulation in *C. barbinervis*, the dynamics of sulfur (S) in response to Ni and Co was discussed mainly.

## 2. Results

### 2.1. Plant Growth and Photosynthesis

**Figure 1:**
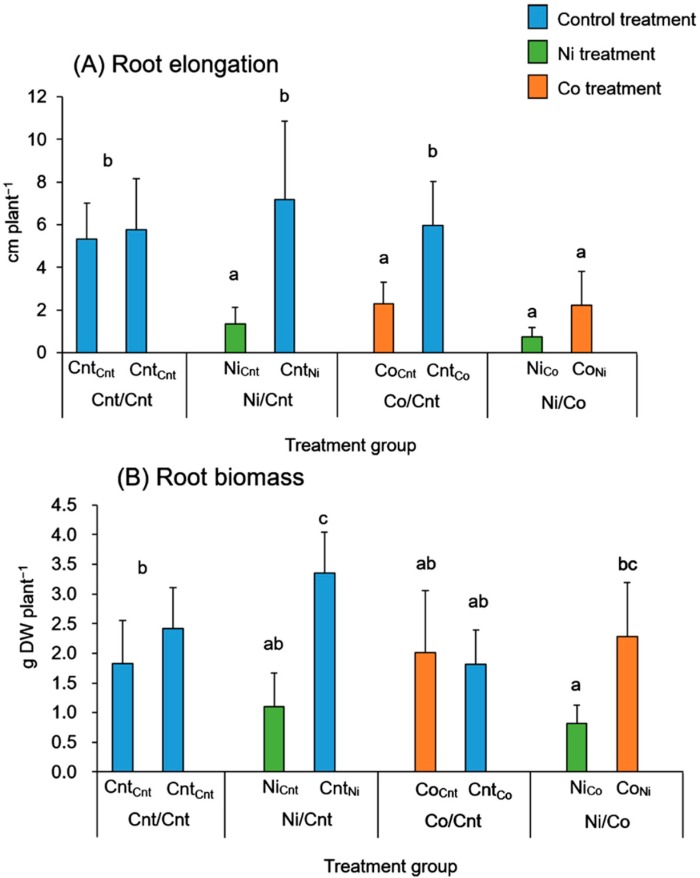
Effect of Ni and/or Co treatments on (**A**) root elongation (cm) and (**B**) root biomass (dry weight (g); DW). Four factorial combinations of the root treatments were conducted; Control solution/Control solution (Cnt/Cnt), Ni solution/Control solution (Ni/Cnt), Co solution/Control solution (Co/Cnt), and Ni solution/Co solution (Ni/Co). Each half of the root system was named with a subscript abbreviation denoting the treatment applied to the other half: Cnt_Cnt_, Ni_Cnt,_ Cnt_Ni_, Co_Cnt_, Cnt_Co_, Ni_Co_, and Co_Ni_. Error bars show SD (*n* = 5, 6). Different letters (a, b and c) above each bar indicate a significant difference (Tukey’s honest significant difference test, *p* < 0.05).

The roots of a seedling were split into two parts and immersed in each solution. Four factorial combinations of root treatments were conducted: Control solution/Control solution (Cnt/Cnt), Ni solution/Control solution (Ni/Cnt), Co solution/Control solution (Co/Cnt), and Ni solution/Co solution (Ni/Co). Each half of the root system was named with a subscript abbreviation denoting the treatment applied to the other half: Cnt_Cnt_, Ni_Cnt,_ Cnt_Ni_, Co_Cnt_, Cnt_Co_, Ni_Co_, and Co_Ni_. In *C. barbinervis,* the elongation of roots treated with Ni or Co solutions, shown as Ni_Cnt_, Ni_Co_, Co_Cnt_, and Co_Ni_, was significantly suppressed compared with the elongation of roots treated with the control solution (Figure 1A). Although the Ni treatment significantly decreased the root biomass from that of the control, another root fragment of each Ni treatment (Cnt_Ni_ and Co_Ni_) tended to have a higher biomass (Figure 1B). Each Co treatment did not affect the root biomass (Co_Cnt_ and Co_Ni_). Table 1 shows the leaf biomass and photosynthetic activity after 7 weeks of treatment. The data indicate that the Ni and/or Co treatments in the rhizosphere did not affect these parameters aboveground.

**Table 1:** Effect of Ni and/or Co treatments on leaf biomass (dry weight (g); DW) and net assimilation rate of CO_2_.

Treatment	Leaves Biomass	Net Assimilation Rates
	g Plant^−1^ DW	µmol m^−2^ s^−1^
Cnt/Cnt	8.11 ± 2.08	7.38 ± 2.6
Ni/Cnt	6.38 ± 1.18	6.47 ± 2.4
Co/Cnt	7.72 ± 0.59	7.55 ± 1.8
Ni/Co	6.13 ± 2.26	7.22 ± 2.2

Values are mean ± SD (*n* = 5, 6). There were not significant differences (Tukey’s honest significant difference test, *p* < 0.05).

### 2.2. Ni, Co, and S Accumulation in Roots and Leaves

**Figure 2:**
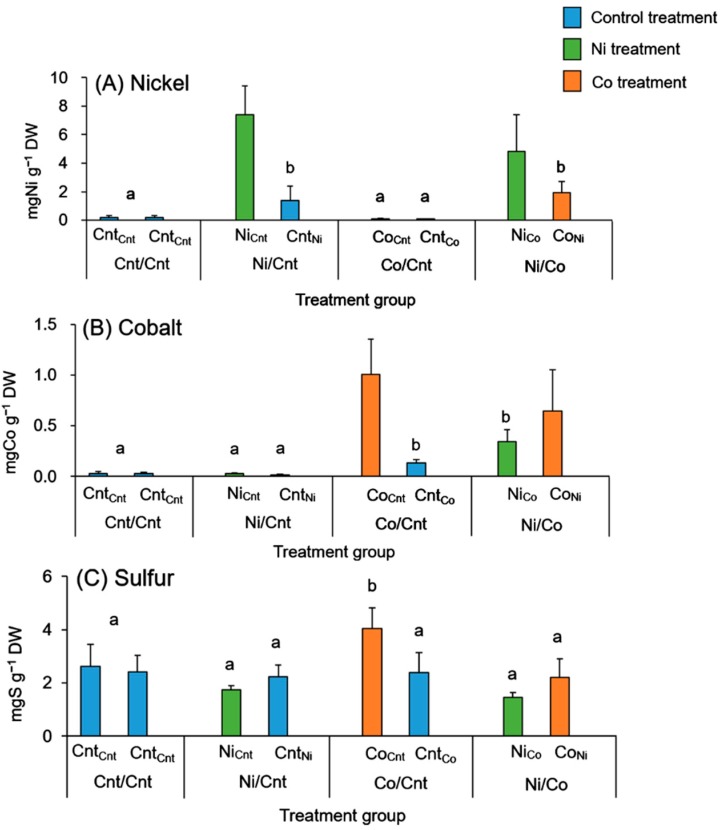
Concentrations of (**A**) nickel (Ni), (**B**) cobalt (Co), and (**C**) sulfur (S) in roots treated with Ni and/or Co. Four factorial combinations of the root treatments were conducted; Control solution/Control solution (Cnt/Cnt), Ni solution/Control solution (Ni/Cnt), Co solution/Control solution (Co/Cnt), and Ni solution/Co solution (Ni/Co). Each half of the root system was named with a subscript abbreviation denoting the treatment applied to the other half: Cnt_Cnt_, Ni_Cnt,_ Cnt_Ni_, Co_Cnt_, Cnt_Co_, Ni_Co_, and Co_Ni_. Error bars show SD (*n* = 5,6). Statistical analyses were conducted between the roots directly treated with Ni or Co (Tukey’s honest significant difference test) and between the other roots (Student’s *t*-test) in Ni and Co concentration. In S, statistical analyses were conducted between all roots (Tukey’s honest significant difference test). Different letters (a, b) above each bar indicate significant differences (*p* < 0.05).

We compared the Ni and Co concentration in the roots by considering the Ni, Co, or control treatments either directly or indirectly. A comparison of the roots receiving direct Ni treatments (Ni_Cnt_ and Ni_Co_) showed that their Ni concentrations were not significantly different (Figure 2A). Similarly, the concentration of Co in the roots was not significantly different between the direct Co treatments (Co_Cnt_ and the Co_Ni_) (Figure 2B). The roots on the other side of the metal-treated roots, roots without Ni or Co treatments, also contained the respective metals at much higher concentrations than roots without any Ni or Co treatment (the roots of Cnt/Cnt and Co/Cnt for Ni accumulation, and the roots of Cnt/Cnt and Ni/Cnt for Co accumulation; Figure 2A,B).

The treatments with Ni and/or Co solutions in the rhizosphere resulted in highly-accumulated metals in the plant leaves (Table 2). The leaves of plants treated with Ni and/or Co accumulated each corresponding element: Ni: 116.2–483.8 µg g^−1^ dry weight (DW) and 160.0–749.1 µg g^−1^ DW for the Ni/Cnt and Ni/Co treatments, respectively; and Co: 242.3–1102 µg g^−1^ DW and 368.1–1102 µg g^−1^ DW for the Co/Cnt and Ni/Co treatments, respectively. The Ni- and Co-treated seedlings (Ni/Co) accumulated both metals not only in their leaves, but also in their bark and wood at high concentrations (Table 2). There were no differences in the Ni and Co concentration in leaves from the plants treated with Ni/Cnt or Co/Cnt and those with Ni/Co, although the Co concentration in the barks and woods of the Ni/Co treatment group was significantly higher than that of the Co/Cnt treatment group. By comparing the concentrations of Ni and Co among various tissues, we observed a similar order, *i.e.*, roots > leaves > bark > wood for Ni, and roots ≥ leaves > bark > wood for Co.

The S concentrations in the roots and leaves of the Co/Cnt treatment group were significantly higher than those of the Cnt/Cnt and Ni/Cnt treatment groups. Furthermore, the S concentrations in the leaves of the Ni/Co treated plants tended to be higher than the S concentrations in the leaves without the Co treatments (Cnt/Cnt and Ni/Cnt; Figure 2C, Table 2). In the bark and wood, the S concentration did not significantly differ between the Ni or Co treatment groups (Table 2).

**Table 2:** Element concentrations in each tissue of *Clethra barbinervis* treated with Ni and/or Co.

Plant Tissues	Treatment	Ni	Co	S
		µg g^−1^ DW	µg g^−1^ DW	µg g^−1^ DW
Leaves	Cnt/Cnt	0.81 ± 0.07 a	0.16 ± 0.12 a	2240 ± 632 a
	Ni/Cnt	257 ± 134 b	0.83 ± 0.69 a	1890 ± 357 a
	Co/Cnt	1.30 ± 0.74 a	697 ± 324 b	4040 ± 1510 b
	Ni/Co	411 ± 263 b	635 ± 257 b	2820 ± 883 ab
Barks	Cnt/Cnt	3.62 ± 2.98 a	2.15 ± 0.52 a	904 ± 241 a
	Ni/Cnt	117 ± 65.0 b	0.58 ± 0.43 a	686 ± 82.4 a
	Co/Cnt	3.16 ± 0.91 a	50.6 ± 7.07 b	792 ± 113 a
	Ni/Co	151 ± 92.6 b	81.5 ± 32.9 c	844 ± 136 a
Woods	Cnt/Cnt	2.70 ± 0.68 a	0.53 ± 0.34 a	752 ± 208 a
	Ni/Cnt	42.3 ± 17.2 b	0.13 ± 0.22 a	704 ± 114 a
	Co/Cnt	2.41 ± 1.23 a	11.8 ± 2.95 b	751 ± 275 a
	Ni/Co	47.3 ± 15.1 b	19.5 ± 6.06 c	850 ± 200 a

Values are the mean ± SD (*n* = 5, 6). Different letters (a, b, c) within a column indicate a significant difference (Tukey’s honest significant difference test, *p* < 0.05). DW, dry weight.

### 2.3. Comparison between Ni and Co Accumulation Using BCF

**Figure 3:**
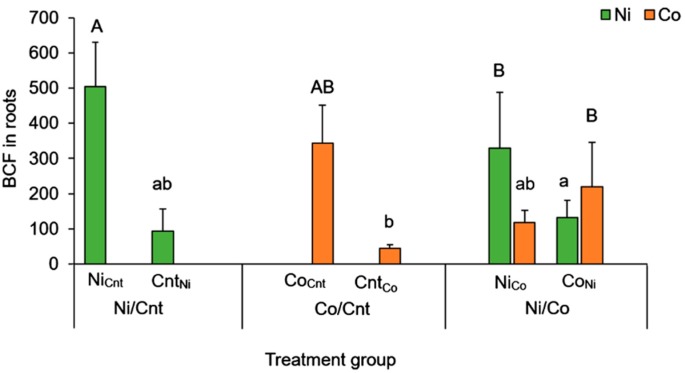
Bioconcentration factors (BCFs) of Ni and Co in roots treated with Ni and/or Co. Green bars show the BCFs for Ni and orange bars show those for Co. Three factorial combinations of the root treatments were conducted; Control solution/Control solution (Cnt/Cnt), Ni solution/Control solution (Ni/Cnt), Co solution/Control solution (Co/Cnt), and Ni solution/Co solution (Ni/Co). Each half of the root system was named with a subscript abbreviation denoting the treatment applied to the other half: Ni_Cnt,_ Cnt_Ni_, Co_Cnt_, Cnt_Co_, Ni_Co_ and Co_Ni_. Error bars show SD (*n* = 5–6). Statistical analyses were conducted between the roots directly treated with Ni or Co (significant difference at *p* < 0.05 was shown with A and B), and between non-treated roots with Ni or Co (difference was shown with a and b) (Tukey’s honest significant difference test).

we used the BCF to compare the accumulation efficiency of Ni and Co in the tissues (Figure 3, Table 3) because the concentrations of Ni and Co in the treatment solutions were different (Ni: 250 µM, Co: 50 µM). There were six types of heavy-metal treatments applied to the roots, excluding the Cnt/Cnt treatment, which we compared by dividing the treatments into two groups: one with and one without the direct heavy-metal treatment. The first group consisted of Ni_Cnt_ and Ni_Co_ for Ni accumulation, and Co_Cnt_ and Co_Ni_ for Co accumulation. The second group, which represented the other half of the split roots treated with Ni or Co solutions, consists of Co_Ni_, and Cnt_Ni_ for Ni accumulation and Cnt_Co_ and Ni_Co_ for Co accumulation. The second group examined the translocation efficiency of Ni or Co from the roots treated with the Ni or Co solution. For the same elements, the BCF tended to differ between plants receiving single heavy-metal treatments (Ni/Cnt and Co/Cnt) and those receiving multiple heavy-metal treatments (Ni/Co). However, we could not clearly confirm these differences in the BCF of Ni and Co. This means that the uptake efficiency from the surrounding solution to inside the roots did not differ between Ni and Co. The BCFs of Co tended to be higher than those of Ni in the wood and bark and were markedly higher than those of Ni in the leaves.

**Table 3:** Bioconcentration factors (BCFs) in each tissue of *Clethra barbinervis* treated with Ni and/or Co.

Element	Treatment	BCF		
		Leaves	Barks	Wood
Ni	Ni/Cnt	17.5 ± 8.36 a	8.01 ± 4.05 a	2.88 ± 1.05 a
	Co/Cnt	-	-	-
	Ni/Co	28.0 ± 16.4 a	10.3 ± 5.76 a	3.22 ± 0.94 a
Co	Ni/Cnt	-	-	-
	Co/Cnt	236 ± 100 b	17.2 ± 2.19 ab	4.00 ± 0.91 a
	Ni/Co	216 ± 79.5 b	27.6 ± 10.2 b	6.62 ± 1.88 b

Values are mean ± SD (*n* = 5, 6). Different letters within a column indicate a significant difference (Tukey’s honest significant difference test, *p* < 0.05). Hyphen means no value.

## 3. Discussion

### 3.1. Effects of Ni and Co on Root Growth

Ni and Co are known as mitotic inhibitors and they reduce root growth [6,18]. The inhibition effect of Ni and Co on root elongation should appear in the roots treated with Ni or Co; however, we found that the roots treated with Co may have a strategy to maintain their root biomass, probably through thickening or branching of their roots. These results suggest that the roots of *C. barbinervis* can absorb Ni and Co at a same rate but the function of the incorporated Ni and Co within the roots is different.

### 3.2. Difference between Ni and Co Accumulation and Translocation

In the leaves, the BCF for Co was significantly higher than that for Ni (Table 3), and the results indicate that Co was transported from the root to the leaves more efficiently than Ni. The differences between ^109^Cd, ^57^Co, ^65^Zn, and ^63^Ni uptake, transport, and redistribution have been determined in white lupin (*Lupinus albus* cv. Amiga) [19], wheat (*Triticum aestivum* L. cv. Arina) [20,21], and the Cd-hyperaccumulator *Solanum nigrum* L [22]. These studies showed that the high mobility of Ni and Zn in plants and the accumulation of Co in roots are common phenomena. Wei *et al*. reported that the mobility of Cd in *S. nigrum* L was higher than that in lupin and wheat, which retained Cd in its roots and released it slowly to the shoots [22]. This indicates that metal-accumulator plants have special properties for the transport and accumulation of specific metals. Our results for *C. barbinervis* show a phenomenon that is contrary to the previous studies on Co mobility. We suggest that *C. barbinervis* has a specific mechanism for accumulating or transporting Co, which enables Co to be transported more efficiently than Ni.

Elevated concentrations of Ni in plant tissues are known to inhibit photosynthesis and decrease biomass [6,23]. Co toxicity also includes marked decreases in shoot biomass and chlorophyll content via inhibition of photosynthesis in various plant species [7,24]. In the current study, we observed high Ni and/or Co accumulation in the leaves (116–749 µg g^−1^ for Ni and 242–1102 µg g^−1^ for Co; Table 2) without any effect on photosynthesis or leaf biomass (Table 1). Therefore, we propose that *C. barbinervis* has a detoxification mechanism for Ni and Co in its leaves.

The leaves of *C. barbinervis* accumulated Ni or Co at high and similar concentrations in each treatment group (Table 2). We also found the simultaneous accumulation of Ni and Co by the Ni/Co treatment at high concentrations similar to those for the single treatments (Ni/Cnt and Co/Cnt). These results suggest that the accumulation and detoxification mechanisms for Ni and Co are different and act separately for each element in the leaves of *C. barbinervis*. Since the free ion species is the most cytotoxic in the case of heavy metals, chemical binding with various chelating substances such as phytochelatins or organic acids and intracellular sequestration into vacuoles are well-known detoxification mechanisms [17,25,26,27]. If plants accumulate or transport several metals with the same detoxification mechanisms, the accumulation of each metal can show a competitive phenomenon. Küpper *et al*. demonstrated that Cd uptake is reduced by exposure to Zn, suggesting a common pathway due to their chemical similarities [28]. Co frequently interacts antagonistically with Ni, Fe, and Mn in plants [6,7], and competitive interactions between Ni and Co have been observed in *A. bertolonii* [8] and *B. coddii* [9]. On the other hand, *Alyssum murale* can accumulate Ni and Co simultaneously by utilizing different accumulation mechanisms for each metal [17]. These findings support the proposal that *C. barbinervis* may have different accumulation and detoxification mechanisms for Ni and Co and that it may enable the simultaneous accumulation of Ni and Co in the leaves without any adverse effects.

*C. barbinervis* accumulated high concentrations of Ni or Co in the roots treated with each element as well as in the other half of the roots that were not treated with Ni or Co directly (Figure 2A,B). The metal concentrations in the directly treated roots were higher than in the roots on the other side, suggesting that *C. barbinervis* transported and accumulated Ni or Co to the roots on the other side. Welch *et al*. reported similar results for Cd and Zn translocation using a split-root experimental system [10]. They suggested that Cd movement from Cd-treated roots to non-treated roots must occur via the phloem, either directly through phloem connections between root segments or secondarily by remobilization of Cd from the shoot [10]. Haslett *et al*. suggested that translocation to other roots within the same plant occurs through phloem connections between root segments [29]. However, Page and Feller suggested that metals translocated to leaves from one part of a root system were then retranslocated from those leaves to other parts of the root system [20]. In the present study, we observed that Ni concentrations in the leaves correlated with Ni concentrations in Cnt_Ni_ roots (*r* = 0.90, *p* < 0.05) in the Ni/Cnt treated group. In this case, it was considered that Ni was transported to the roots on the other side via the leaves. On the other hand, no such relationship was found in the Co/Cnt treatment group. This result was supported by the high BCF value for Co in the leaves, indicating that Co tends to accumulate in this tissue.

### 3.3. Relationship between S and Co

The majority of the S in a plant exists in the form of inorganic sulfate anions, and the rest of the S is incorporated into organic compounds such as proteins, chloroplast lipids, or secondary metabolites [30,31]. S is known as both structural and functional constituent of proteins, namely cysteine and the strong antioxidant glutathione [30]. Additionally, S is strongly related to the detoxification of heavy metals such as by phytochelatins [31]. We found higher concentrations of S in the leaves of the Co/Cnt treated group than in the those of the other groups (Table 2), and the concentration of S significantly correlated with that of Co (*r* = 0.85, *p* < 0.05) in leaves. This result indicates that S was related to Co accumulation in the leaves. Oven *et al.* suggested that free cysteine is involved in Co-ion complexation in plant cells [32]. Tappero *et al*. reported that Co accumulation associated with S in the leaves of the hyperaccumulator *A. murale*. They found that elevated S concentrations were correlated with those of Co, based on the SXRF maps, and they suggested that S acts as a counter ion that is required for charge balance against Co [17]. In case of roots, we found that the elevated S concentration in Co_Cnt_ roots had no correlation with Co concentration.

The accumulation mechanisms related to S via cysteine and glutathione or by the counter-ion role of S were also reported for Ni accumulation in *Thlaspi* [33] and *Alyssum* species [15,16]. Although S has a similar relationship to Ni and Co accumulation in several accumulator plants, *C. barbinervis* shows a relationship with S only for Co accumulation and not for that for Ni (Table 2). Tappero *et al*. reported similar results regarding the difference between Ni and Co using *A. murale*, indicating that S was related to only Co detoxification [17]. The published results and those of our study indicate that S plays a role in Co transport to the leaves from the roots and Co accumulation in the leaves. The distinction between Ni and Co in *C. barbinervis* may occur through the reaction of some specific S species with Co.

### 3.4. Interaction between Ni and Co

We have reported several properties of *C. barbinervis* regarding transport and accumulation of Ni and Co upon single-metal treatment (Ni/Cnt and Co/Cnt), in which Ni accumulation in the roots relates to that in the leaves and to the relationship between Co and S concentrations. In addition, we found that *C. barbinervis* may have different accumulation and detoxification mechanisms for Ni and Co, which enable the simultaneous accumulation of Ni and Co in the leaves in the Ni/Co treated plants. The results for the Ni/Co treatment group differed from those of the single-treatment group. We did not find any correlation between S and Co in the leaves of the Ni/Co-treated seedlings, suggesting that high Ni accumulation together with Co is related to S. Otherwise, for Ni accumulation, regarding the contribution of organic acids and amino acids, such as citric acid, malic acid, and histidine, these have been known as ligands in the tissues of several plants [18]. Such ligands acting to accumulate Ni may also act as ligands for Co accumulation in the Ni/Co-treated-plant leaves. This may be another reason for the lack of a relationship between S and Co in the Ni/Co leaves.

The BCF results in the roots also show the different trends between the single treatment (Ni/Cnt and Co/Cnt) and the multiple treatments (Ni/Co) groups. The BCFs in the roots of Ni_Cnt_ and Co_Cnt_ that were treated with Ni or Co directly with the single-metal treatment were higher than those in the roots of Ni_Co_ and Co_Ni_ in the multiple-metal treatments. On the other hand, the BCFs in Ni_Co_ and Co_Ni_ were higher than those in Cnt_Ni_ and Cnt_Co_, which were the indirectly treated roots in the single treatment (Figure 3). These results indicate that Ni and Co in the Ni/Co treatment groups were transported more efficiently than those in the Ni/Cnt- and Co/Cnt-treated plants, suggesting that a kind of synergistic effect exists for the simultaneous transport of Ni and Co. Furthermore, the BCFs of Co in the bark and wood of the Ni/Co-treated seedlings tended to be higher than in those in the corresponding tissue of the Co/Cnt-treated seedlings due to a kind of a synergistic relationship (Table 3). Synergistic interactions have been recognized as an upregulation of the uptake or transport mechanism for multiple elements caused by many transmembrane metal transporters involved in a variety of processes in plants [34]. In the Ni/Co-treated seedlings, each transport mechanism related to Ni or Co may be regulated simultaneously. If each mechanism for Ni and Co has an affinity for another metal, the transport and accumulation of Ni and Co could occur under the coexistence of these two elements without their competition. Therefore, the Ni/Co-treated seedlings may transport Ni and Co in the roots, bark, and wood more efficiently than seedlings treated with single elements.

## 4. Experimental Section

### 4.1. Experimental Design and Plant Growth

**Figure 4:**
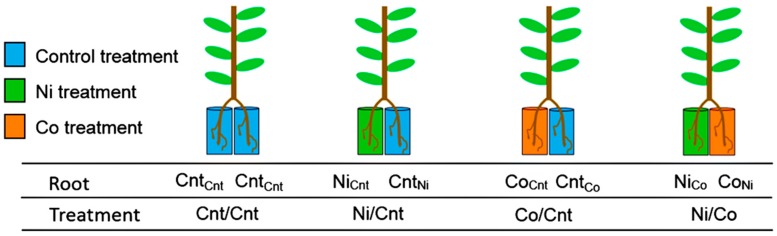
Treatment combinations for the split-root experiment. Blue, green, and red pots show that the pots contain Control solution, Ni solution, and Co solution, respectively. The roots of a seedling were split into two parts and immersed in each solution. Four factorial combinations of the root treatments were conducted; Control solution/Control solution (Cnt/Cnt), Ni solution/Control solution (Ni/Cnt), Co solution/Control solution (Co/Cnt), and Ni solution/Co solution (Ni/Co). Each half of the root system was named with a subscript abbreviation denoting the treatment applied to the other half: Cnt_Cnt_, Ni_Cnt,_ Cnt_Ni_, Co_Cnt_, Cnt_Co_, Ni_Co_, and Co_Ni_.

Seedlings of *C. barbinervis* were used to measure growth, photosynthesis, and metal uptake in response to elevated Ni and Co concentrations in hydroponic culture. Seedlings of *C. barbinervis* were purchased from Kumamoto ryokka center in Kumamoto prefecture, Japan. The roots were washed to remove soil and the plants were transferred to plastic pots filled with tap water and grown for 1 month until the treatment started. Three kinds of nutrient solutions, including the control treatment, the Ni treatment, and the Co treatment, were prepared based on 1/10 Hoagland’s No.2 solution. The Ni and Co solutions were prepared by adding NiSO_4_·6H_2_O or CoCl_2_·6H_2_O to the base solution to attain 250 µM Ni and 50 µM Co, respectively. In order to adjust the concentrations of SO_4_^2−^ in the Co and control solutions so that they were equivalent to the Ni solution, Na_2_SO_4_ was added to the control and Co solutions to attain 250 µM SO_4_^2−^. The difference in the magnitude of the Ni (250 µM) and Co (50 µM) solutions relates to the concentrations detected in the soil solution of serpentine soil (extracted by 0.1 M HCl) (Shinshiro, Japan.) For one seedling, two small boxes were prepared. The roots of a seedling were split into two parts and immersed in each solution. Four factorial combinations of root treatments were conducted: Control solution/Control solution (Cnt/Cnt), Ni solution/Control solution (Ni/Cnt), Co solution/Control solution (Co/Cnt), and Ni solution/Co solution (Ni/Co) (Figure 4). The seedlings were grown in a greenhouse under natural environmental conditions from 12 June 2013. The average day/night temperatures were 28 °C/20 °C. The treatment solutions were renewed once every 10 days. During the treatments, the solution level in each box was adjusted to the initial level.

### 4.2. Plant Tissue Analyses

After 21 weeks of treatments, the seedlings were harvested and separated into the roots, leaves, bark and wood. The roots were washed with deionized water using an ultrasonic bath for less than 1 min, rinsed with 0.1 M ethylenediaminetetraacetic acid solution to remove surface metal elements, and then rinsed with deionized water. The other plant parts were washed with deionized water. All samples were dried at 80 °C for 48 h, and the dried samples were weighed to determine their biomass. Approximately 0.1 g of dried plant sample was digested with 5 mL of concentrated nitric acid in microwave digestion apparatus (ETHOS 1600, MILESTONE, Sorisole, Italy). The digest was diluted to 25 mL with deionized water and filtered with a 0.45 µm membrane filter. The sample solution was analyzed by inductively-coupled plasma atomic emission spectrometry (ICP-AES; IRIS ICARP, Jarrell Ash Nippon Corp., Kyoto, Japan).

The root elongation was represented by the maximum length and was measured before and after the treatment. The difference in root length before and after the treatment was defined as the root extension value.

### 4.3. Calculation of BCF

Since the concentrations of Ni and Co in the treatment solutions were different (Ni: 250 µM, Co: 50 µM), we calculated the BCF to compare the accumulation efficiency of Ni and Co in the tissue.

(1) $$\text{BCF}=\frac{\text{metal concentrations of each tissues}}{\text{metal concentrations of each treatment solutions}}$$

### 4.4. Measurement of Photosynthesis

To determine the photosynthetic activity, the net CO_2_ assimilation rates of undamaged, matured leaves were measured using a LI-6400 Portable Photosynthesis System (LiCor Inc.: Lincoln, NE, USA). To determine the light saturation point of the net assimilation rate, the net CO_2_ assimilation rates with varying PAR (0 to 1500 µmol m^−^^2^ s^−1^) were measured using the artificial light source of the LI-6400 and the leaves of several seedlings from each treatment group. From the results, PAR 700 µmol m^−2^ s^−1^ was determined as the light saturation point. We measured the net assimilation rate for each tree at this light condition. The measurements were conducted on the day 7 weeks after the treatment started.

### 4.5. Statistical Analyses

Differences in the data between each treatment group were assessed using Student’s *t*-tests or by one-way ANOVA followed by Tukey’s honest significant difference multiple comparison test (*p* < 0.05). In addition, Pearson’s correlation coefficients were calculated to assess correlations between the concentrations of Ni or Co and S. Similarly, Pearson’s correlation coefficient was calculated for the Ni or Co concentrations in the roots and leaves were calculated. For these analyses, we used the free statistical software R, version 2.15.1 (R Development Core Team 2012, Vienna, Austria).

## 5. Conclusions

We hypothesized that *C. barbinervis* absorbs and accumulates Ni and Co without distinction. To test this hypothesis, we conducted a split-root experiment using Ni and/or Co solutions. We found that the uptake and accumulation efficiency of Ni were similar to those of Co in the roots, but the transport and accumulation efficiency in the leaves was higher for Co than for Ni. This indicates that the roots of *C. barbinervis* can absorb Ni and Co at a same rate, but that *C. barbinervis* has a specific mechanism to accumulate or transport Co in the leaves that differs from that for Ni. We also found that S was elevated in the roots and leaves of the seedlings treated with Co, indicating that S is involved in the Co accumulation mechanism. The seedlings simultaneously treated with Ni and Co tended to transport Ni and Co in the roots, bark, and wood more efficiently than the single element treatments. The results suggest that the transport and accumulation of Ni and Co occur under the coexistence of these two elements without their competition. In conclusion, *C. barbinervis* can distinguish Ni and Co during transport and accumulation in the leaves but not during root uptake.
